# TMEM16A overexpression contributes to tumor invasion and poor prognosis of human gastric cancer through TGF-β signaling

**DOI:** 10.18632/oncotarget.3412

**Published:** 2015-03-23

**Authors:** Fang Liu, Qing-Hua Cao, De-Jian Lu, Bin Luo, Xiao-Fang Lu, Rong-Cheng Luo, Xiao-Guang Wang

**Affiliations:** ^1^ Department of Oncology, Nanfang Hospital, Southern Medical University, Guangzhou 510515, China; ^2^ Faculty of Forensic Medicine, ZhongShan School of Medicine, Sun Yat-sen University, Guangzhou 510080, China; ^3^ Cancer Center and Traditional Chinese Medicine-Integrated Hospital, Southern Medical University, Guangzhou 510315, China; ^4^ Department of Pathology, The First Affiliated Hospital of Sun Yat-sen University, Guangzhou 510080, China

**Keywords:** TMEM16A, invasion, prognosis, gastric cancer, TGF-β

## Abstract

TMEM16A is a newly identified calcium activated chloride channel, and has been reported to be overexpressed by various solid malignant cancers to promote proliferation and invasion, yet little is known about its role in gastric cancer(GC). Therefore, we investigated the role of TMEM16A in GC and its clinical significance by a retrospective analysis of 367 GC patients, and *in vitro* study was performed for validation and underlying molecular mechanism.

TMEM16A was significantly upregulated and amplified in GC tissues, and its overexpression was positively correlated with disease stage, negatively with patient survival and identified as an independent prognostic factor for patient outcome. A negative correlation between TMEM16A and E-cadherin was found in 367 GC specimens. TMEM16A silencing significantly decreased calcium activated chloride currents, impaired TGF-β secretion, reduced E-cadherin expression, and inhibited the migration and invasion without affecting proliferation of GC cells (AGS and BGC-823). Supplement of TGF-β reverted the effects of TMEM16A silencing on E-cadherin expression, cell migration and invasion.

In conclusion, TMEM16A promotes invasion and metastasis in GC, and might be a novel prognostic biomarker and potential therapeutic target in the treatment of GC.

## INTRODUCTION

Gastric cancer (GC) is the fourth most common cancer and the second most common cause of cancer-related death worldwide [1], especially the incidence of GC is much higher in china than in any other country [2]. Although the diagnosis, staging and treatment of GC have improved over past decades, the prognosis remains poor due to local invasion and distal metastasis [3]. The complex molecular mechanisms underlying invasion and metastasis are not well characterized [4].

Transmembrane protein 16A (TMEM16A) also known as ANO1, DOG1 or TAOS2, was identified as a novel component of calcium activated chloride channels (CaCC) [5]. Recently, TMEM16A has been found to be upregulated in many tumor types including gastrointestinal stromal tumor, breast cancer, prostate cancer, esophyageal cancer, and head and neck squamous cell cancer (HNSCC) [6–9], and overexpression of TMEM16A has been implicated in promoting tumor proliferation [10], migration and invasion [11]. However, the biological role and clinical significance of TMEM16A in GC remain largely elusive. Furthermore, TMEM16A is located within 11q13 chromosome amplicon, which is proved to be associated with carcinogenesis of GC [12, 13]. All of these information aroused our curiosity to investigate whether TMEM16A is relevant with tumorigenesis and progression of GC.

In this study, we aimed to examine the role and potential mechanisms of TMEM16A in GC by a retrospective analysis of 367 GC patients and 5 mounted section GC tissues, and by carrying out *in vitro* experiments to clarify the impact of TMEM16A on GC proliferation, invasion and potential mechanism. We found that TMEM16A was markedly upregulated and amplified in GC tissues, and its overexpression significantly correlated with the clinicopathological characteristics and shorter survival of patients with GC, also its expression was inverse relation with E-cadherin in 367 GC specimens, corresponding lymph node metastases and adjacent no-tumor tissues. Downregulation of TMEM16A abrogated the ability of migration and invasion and promoted E-cadherin expression. We further demonstrated that knockdown TMEM16A inhibited secretion of TGF-β to upregulate E-cadherin expression. Our findings suggest that TMEM16A plays a role in invasion and metastasis in GC, and might be a novel prognostic biomarker and potential therapeutic target in the treatment of patients with GC.

## RESULTS

### TMEM16A is overexpressed in GC tissues and associated with poor prognosis of GC

**Figure 1:**
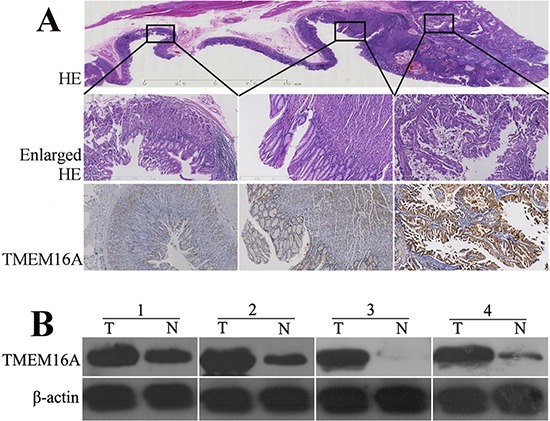
The expression of TMEM16A protein in gastric cancer, adjacent and normal tissues **A.** A representative hematoxylin and eosin (HE) stained mounted section containing gastric cancer, adjacent and normal gastric mucosa (the upper row, original magnification, ×4) with enlarged view(the middle row, original magnification, ×40). The lower row displayed that TMEM16A was strongly expressed in gastric cancer(the right panel), whereas was moderately or weakly expressed in adjacent(the middle panel) and normal tissues(the left panel) (original magnification, ×40). **B.** Western blot analysis of TMEM16A protein expression in four pairs of matched gastric tumor (T) and adjacent non-tumor mucosa (N). Equal loading of protein was determined by β-actin.

To confirm whether TMEM16A was overexpressed in GC, we performed immunohistochemistry (IHC) of mounted sections, western blotting of surgical samples. Expression of TMEM16A was found to be significantly higher in tumor tissues than that in adjacent non-tumor tissues (Fig. 1A, B). Then, TMEM16A protein expression in 367 GC tissues, corresponding lymph node metastatic lesions and normal gastric tissues was assessed by immunohistochemical staining. Consistent with above result, TMEM16A was strong expressed in GC and lymph node metastasis lesions on tissue microarray (TMA), whereas weak expressed in adjacent non-tumor gastric mucosal tissues.

To generate a reasonable cutoff score of TMEM16A protein for further survival analysis, we firstly subjected the IHC scores of TMEM16A protein to operating characteristic (ROC) curve analysis with respect to their overall survival in the training set. We found that the IHC score cutoff point of TMEM16A was 8.5. We thus selected a TMEM16A expression score of 8 (> 8 VS. ≤ 8) as the cutoff point to distinguish the patients as high or low expression for survival analysis in the testing set.

The training set ROC-derived TMEM16A cutoff score of 8 successfully segregated the testing set into high (179/254, 70.5%) and low (75/254, 29.5%) TMEM16A expression subgroups. As shown in Table 1, TMEM16A was significantly correlated with later TNM stage and present status of lymph node metastasis in both training set (*p* = 0.007, *p* = 0.000) and testing set (*p* = 0.033, *p* = 0.001). However, there was no significant association between TMEM16A protein expression and other patient characteristics, including gender, age at surgery, tumor location, tumor size and Lauren classification.

**Table 1:** Correlation of TMEM16A expression with patients' features in gastric cancer

Variables	All cases	TMEM16A protein					
		Training set(*n* = 113)			Testing set(*n* = 254)		
		Low	High	*P* value^a^	Low	High	*P* value^a^
**Gender**
Male	256	22	55	0.096	52	127	0.797
Female	111	16	20		23	52	
**Age at surgery**							
≥57^b^	191	14	34	0.388	37	106	0.147
<57	176	24	41		38	73	
**Tumor location**							
Upper half	193	17	43	0.422	40	93	0.795
Lower half	158	19	28		33	78	
Whole	16	2	4		2	8	
**Tumor size**							
≥5cm^c^	221	24	46	0.850	42	109	0.469
<5cm	146	14	29		33	70	
**Histological type**							
Intestinal	288	26	59	0.233	58	145	0.505
Diffuse	79	12	16		17	34	
**TNM**							
I + II	139	22	19	**0.007**	39	59	**0.033**
III + IV	228	16	56		36	120	
**Lymphnode metastasis**							
Present	249	17	59	**0.000**	40	133	**0.001**
Absent	118	21	16		35	46	

a chi-square test

b median age

c median tumor size

**Figure 2:**
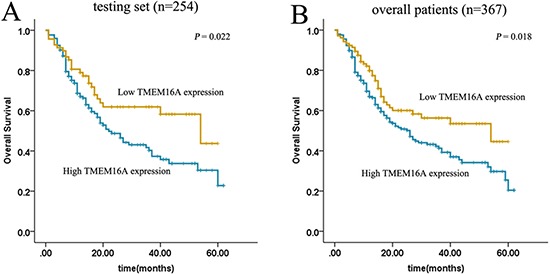
Kaplan–Meier estimated of overall survival according to TMEM16A expression in patients with gastric cancer (log-rank test) High expression of TMEM16A protein was closely correlated with inferior overall survival(OS) in both testing set **A.** and overall patients **B.** (testing set *p* = 0.022; overall patients *p* = 0.018).

By univariate survival analyses using Kaplan–Meier method and log-rank test, the impact of TMEM16A on patient survival was analyzed and we found that elevated expression of TMEM16A was closely associated with poor overall survival in both testing set (*p* = 0.022) and overall patients (*p* = 0.018) (Fig. 2A, 2B).

Multivariate Cox regression analysis was carried out to evaluate the potential prognostic significance of TMEM16A expression and other parameters. In the testing set, TMEM16A was indeed found to be a significant independent prognostic factor for overall survival (hazard ratio, 0.599; 95% confidence interval, 0.381–0.942; *p* = 0.026) (Table 2). Consistently, similar results were also demonstrated in overall patients ((hazard ratio, 0.617; 95% confidence interval, 0.428–0.890; *p* = 0.010) (Table 2).

**Table 2:** Results of multivariate Cox proportional-hazards analysis in testing set and overall patients

Variables	Testing set(*n* = 254)			Overall patients(*n* = 367)		
	*P* value	HR	95% CI	*P* value	HR	95% CI
**Gender**^a^	0.652	1.100	0.726–1.668	0.508	0.887	0.622–1.265
**Age at surgery**^b^	0.968	0.992	0.668–1.473	0.134	0.779	0.561–1.080
**Tumor location**^c^	**0.019**	1.534	1.073–2.193	**0.014**	1.432	1.076–1.904
**Tumor size**^d^	0.926	1.019	0.690–1.503	0.401	1.148	0.832–1.586
**Lauren classificaiton**^e^	**0.014**	1.764	1.122–2.733	**0.001**	1.875	1.303–2.699
**pTNM**^f^	**0.000**	2.296	1.581–3.334	**0.000**	2.147	1.572–2.933
**Lymphnode metastasis**^g^	**0.027**	0.417	0.192–0.905	**0.021**	0.468	0.246–0.891
**TMEM16A**^h^	**0.026**	0.599	0.381–0.942	**0.010**	0.617	0.428–0.890

HR, hazards ratio; CI, confidence interval

a Male vs Female

b ≥ 57 y *v* < 57 y

c upper half vs lower half vs whole stomach

d ≥ 5 cm vs < 5 cm

e intestinal vs diffuse

f I + II vs III + IV

g present vs absent

h low expression vs high expression

### TMEM16A overexpression is more pervasive than amplification

**Figure 3:**
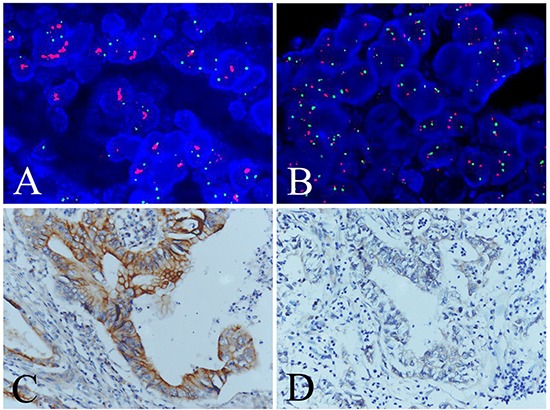
Evaluation of TMEM16A amplification and overexpression in GC tissues **A.** Positive(case 47) and **B.** negative (case 203) TMEM16A amplification evaluated by FISH analysis. **C.** High (case 47) and **D.** low (case 203) TMEM16A expression evaluated by immunohistochemical analysis. Red signals: TMEM16A genes, green signals: chromosome 11 centromere.

To study whether overexpression of TMEM16A resulted from amplification, 367 GC specimens on TMA and cells of AGS and BGC-823 were analysed by FISH. FISH results showed that TMEM16A amplification was 7.4% (27/367) in GC tissues (about nine tenths high-expression, one tenth low-expression TMEM16A) (Fig. 3) and not found in AGS and BGC-823 cells (Data not shown), suggesting that TMEM16A overexpression (254/367, 69.2%) was more pervasive than amplification. Although TMEM16A amplification was significantly correlated with overexpression (*p* = 0.021, Phi = 0.120) (Table S1), TNM stage (*p* = 0.001) and lymph node metastasis (*p* = 0.023) (Table S2), it failed to be a prognostic factor of overall survival of patients with GC (*p* = 0.557) (Fig. S1).

### TMEM16A is elevated in GC cell lines

**Figure 4:**
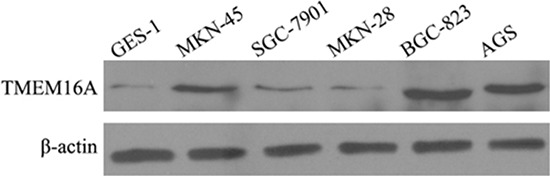
The expression of TMEM16A in GC cell lines by western blot analysis TMEM16A expression was higher in GC cells than that in GES-1, and obviously higher in AGS and BGC-823 cells than that in other GC cells. Equal loading of protein was determined by β-actin.

We examined the expression of TMEM16A in GC cell lines (AGS, MKN-45, BGC-823, SGC-7901, MKN-28). Normal human gastric epithelial cell line (GES-1) was used as reference for TMEM16A expression. Figure 4 showed the expression of TMEM16A was higher in GC cells than that in GES-1, and obviously higher in AGS and BGC-823 cells than that in other GC cells. Therefore, AGS and BGC-823 cells were used to further investigate the influence of TMEM16A on GC.

### Knockdown of TMEM16A does not affect proliferation of GC cells

**Figure 5:**
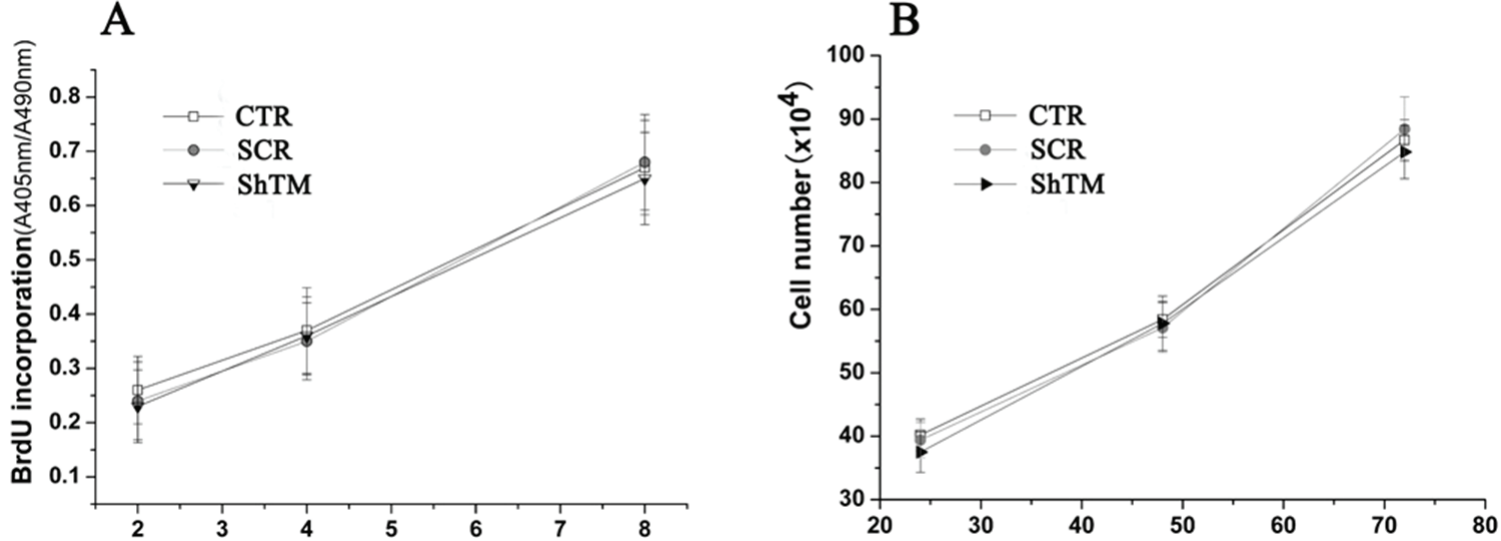
Knockdown of TMEM16A does not affect AGS cell proliferation **A.** BrdU incorporation assay at 2 hours, 4 hours and 8 hours and **B.** cell count experiments at 24 hours, 48 hours and 72 hours from CTR group, SCR group and ShTM group (*n* = 6, *p* > 0.05).

Firstly, we investigated the influence of TMEM16A on AGS cells growth by BrdU incorporation and cell count. BrdU incorporation increased time-dependently in AGS cell lines, which was not affected by knockdown of TMEM16A with short hairpin RNA lentivirus (Fig. 5A). The effects of TMEM16A knockdown on GC cells proliferation were further confirmed by cell count experiments, and at all three time points tested, the cell numbers from CTR group, SCR group and ShTM group were not significantly different (Fig. 5B). These results indicated that ShTM cells proliferated normally and showed no loss of cell viability. Similar to AGS cells, knockdown of TMEM16A did not affect proliferation of BGC-823 cells (Fig. S2A, S2B).

### Knockdown of TMEM16A dramatically inhibits migration and invasion of GC cells

**Figure 6:**
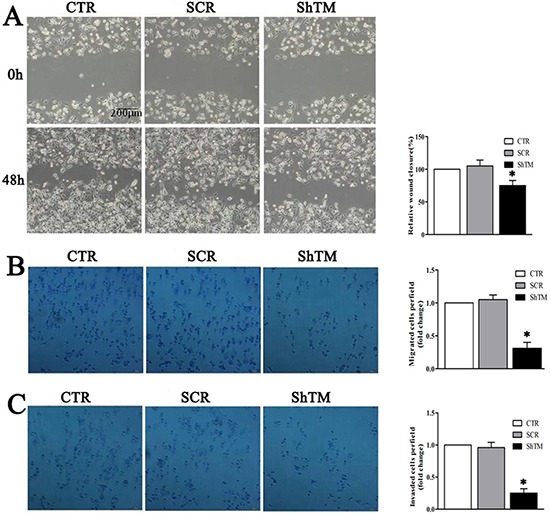
Knockdown of TMEM16A prevents AGS cells migration and invasion **A.** Wound closure assay as shown in phase contrast microscopy images. After 48 h, the wound was nearly closed in CTR and SCR groups. However, knockdown of TMEM16A delayed wound closure significantly though an obvious proliferation was seen in all three groups (*n* = 6, *p < 0.05 vs CTR group). **B.** In the transwell assay, migrated cells significantly reduced after TMEM16A knockdown (*n* = 6, *p < 0.05 vs CTR group). **C.** In the transwell invasion assay, invasiveness was quantified by cells through Matigel and it was showed fewer cells in ShTM group than CTR group and SCR group (*n* = 6, *p < 0.05 vs CTR group). CTR: control AGS, SCR: scrambled AGS, ShTM: shTMEM16A AGS.

We found TMEM16A expression positively correlated with lymph node metastasis status and TNM stage. We further investigated whether TMEM16A interfered with potential of migration and invasion of AGS cells. Cell migration was assessed by two-dimensional wound healing assays and three-dimensional transwell assays. In wound healing assays shown in Fig. 6A, wound closure of SCR group was not significantly different from the CTR group. However, knockdown of TMEM16A reduced the wound-healing ability as wound closure of ShTM group was relatively smaller than that of SCR group. Interestingly, cells from all three groups proliferated fast similarly, and it is expected that cell proliferation should contribute to wound healing except cell migration. To further confirm the role of TMEM16A in cell migration, we conducted a transwell migration assay, and found that deficiency of TMEM16A expression profoundly interfered with the ability of AGS cells to migrate toward a serum gradient (Fig. 6B). Furthermore, we conducted transwell invasion assays to evaluate the effect of TMEM16A on cancer cell invasion and found that knockdown of TMEM16A significantly (*p* < 0.05) reduced the ability of AGS cells to invade through the Matrigel matrix and the number of AGS cells from ShTM group that invaded through the Matrigel matrix was significantly decreased as compared with control (Fig. 6C). Similar results were found in BGC-823 cells (Fig. S3A, S3B).

### TMEM16A expression is negatively related with E-cadherin expression in GC tissues and cells

**Figure 7:**
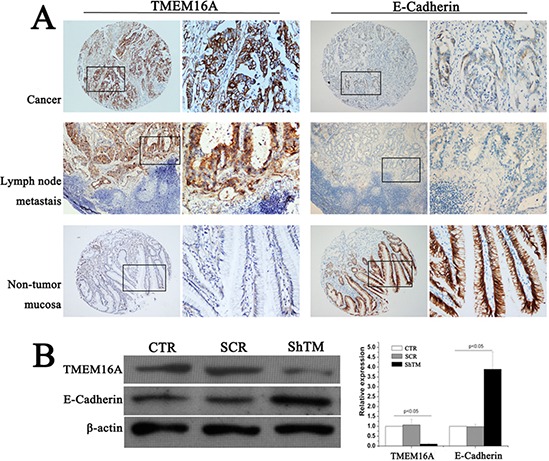
TMEM16A represses E-Cadherin expression **A.** Representative images of TMEM16A and E-cadherin immunostaining in gastric cancer, lymphnode metastasis lesion and non-tumor mucosal tissue (case 125). High expression of TMEM16A protein was detected in gastric cancer (*n* = 367) and lymphnode metastasis lesion (*n* = 30), and low expression was observed in non-tumor mucosal tissue (*n* = 20), but corresponding E-cadherin expression demonstrated the converse results. In panel TMEM16A and E-cadherin, the right panels displayed representative TMEM16A and E-cadherin proteins expression in selected zone with enlarged view. **B.** Knockdown TMEM16A upregulated E-cadherin expression in AGS cells by western blot analysis. Equal loading of protein was determined by β-actin (*n* = 6, *p* < 0.05).

E-cadherin, which forms E-cadherin/catenin complex and then is further linked to the actin cytoskeleton, plays a major role in epithelial cell-cell adhesion [14]. Downregulation of E-cadherin plays a vital role in the initiation and development of GC [15]. We then tested whether knockdown TMEM16A affected E-cadherin expression. First, E-cadherin expression was examined by immunohistochemistry on GC TMA. High E-cadherin expression was detected in 45.5% (167 of 367) GC tissues according to the cutoff point generated by ROC curve analysis. Phiand Cramers V correlation analysis indicated a significantly negative correlation between expression of TMEM16A and E-cadherin in GC (Phi = − 0.161, *P* = 0.002) (Fig. 7A, Table 3). Moreover, similar negative relation was found in lymph node metastases and adjacent non-tumor mucosa tissues (Fig. 7A). Then, subsequent analysis by western blot validated these findings, and protein levels of E-cadherin were significantly increased after TMEM16A knockdown compared with the control group (Fig. 7B).

**Table 3:** The relationship between TMEM16A and E-cadherin expression in gastric cancer tissues by Phiand Cramers V correlation analysis

Variables		All cases	E-Cadherin		*P* value	Phi
			Low(%)	High(%)		
**TMEM16A**	Low(%)	113	48(42.5%)	65(57.5%)	0.002	−0.161
	High(%)	254	152(59.8%)	102(40.2%)		

### TMEM16A suppresses E-cadherin expression through the TGF-β signaling pathway

We noted that TMEM16A was negatively relevant to E-cadherin in GC. How TMEM16A led to the loss of E-cadherin was unknown. We hypothesized that TMEM16A and E-cadherin might have a direct interaction as they are both membrane proteins and related to Ca^2+^ signaling. However, a physical interaction between TMEM16A and E-cadherin was not found by co-immunoprecipitation experiments with anti- TMEM16A and anti-E-caherin antibodies (Data not shown).

Transforming growth factor-β (TGF-β), a multifunctional cytokine, has been shown to induce EMT through repressing E-cadhrerin expression [16]. Low or absent E-cadherin was found in GC by different labs [15, 17]. A recent study found increased expression of TGF-β1 and TGF-β2 both in cancer tissues and serum from GC patients [18]. Several studies supported an important role of TMEM16A in inflammatory response [19–22]. We explored whether TMEM16A downregulated E-cadherin expression by regulating TGF-β signalling.

**Figure 8:**
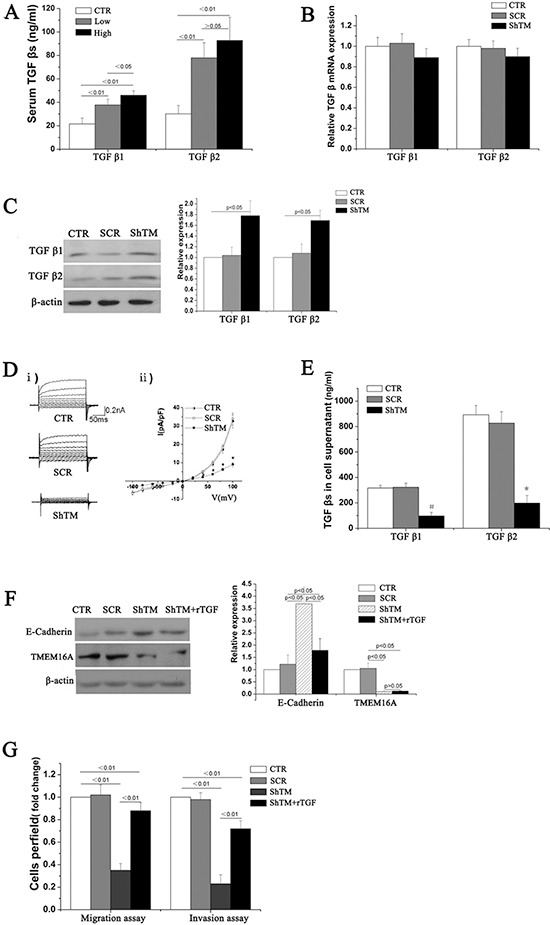
TMEM16A represses E-cadherin expression by modulating the secretion of TGF-β1 and TGF-β2 **A.** Serum levels of TGF-β1 and TGF-β2 was measured by ELISA. Concentrations of TGF-β1 and TGF-β2 were obviously higher in patients with high TMEM16A (*n* = 62) and low TMEM16A expression(*n* = 43) than in healthy volunteers(CTR). **B.** Knockdown of TMEM16A did not affect mRNA expression of TGF-β1 and TGF-β2 in AGS cells(*n* = 6, *p* > 0.05). **C.** Protein expression of TGF-β1 and TGF-β2 in AGS cells increased when knockdown of TMEM16A as shown by western blot analysis(*n* = 6, *p* < 0.05). **D.** Representative Cl^−^ current traces i) and I-V curves ii) of calcium-activated Cl^−^ currents in AGS cells from CTR(*n* = 7), SCR(*n* = 5) and ShTM (*n* = 7) groups (*p < 0.05 vs CTR group). **E.** Supernatant concentrations of TGF-β1 and TGF-β2 dramatically reduced when knockdown of TMEM16A as shown by ELISA(*n* = 6, #*p* < 0.01 vs CTR group, *p < 0.05 vs CTR group). **F.** Adding recombinant purified TGF-β (rTGF-β; R&D Systems) increased E-cadherin expression in AGS cells with TMEM16A knockdown (*n* = 6). **G.** Supplement of recombinant purified TGF-β increased the migration and invasion of AGS cells with TMEM16A knockdown (*n* = 6). CTR: control AGS, SCR: scrambled AGS, ShTM: shTMEM16A AGS.

Serum levels of TGF-β1 and TGF-β2 were firstly measured in 105 patients (43 patients with low-expression and 62 patients with high-expression of TMEM16A, and 30 healthy volunteers). Concentrations of TGF-β1 and TGF-β2 were significantly higher in patients with GC than that in healthy volunteers (Figure 8A), which was consistent with previous researches [18]. Moreover, serum levels of TGF-β1, not TGF-β2, were significantly higher in patients with high-expression of TMEM16A than that with low-expression of TMEM16A (Fig. 8A). Next the effect of TMEM16A on TGF-β expression was investigated in AGS and BGC-823 cells. Surprisingly, knockdown of TMEM16A did not significantly reduce the mRNA expression of TGF-β1 and TGF-β2 (Fig. 8B, S3C), while protein expression of TGF-β1 and TGF-β2 in TMEM16A knowdown cells was significantly increased as shown by western blot (Fig. 8C, S3D). A recent study suggested that TMEM16A served as an important regulator of proinflammatory cytokine secretion [23]. It was suggested that activation of TMEM16A chloride channel regulated cytokine secretion possiblely by depolarization of membrane potentials [24]. We found knockdown of TMEM16A dramatically decreased calcium-activated chloride currents in AGS cells (Fig. 8D). It was reported that TMEM16A contributed to exocytosis and secretion of protein rather than synthesis [25]. Was it possible that TMEM16A regulated TGF-βs secretion? To verify the hypothesis, cell supernatants were collected and concentrations of TGF-β1 and TGF-β2 were measured by sandwich ELISA. TGF-β1 and TGF-β2 levels from the TMEM16A knockdown group were significantly reduced (Fig. 8E, S3E). To further confirm involvement of TGF-β in downregulation of E-cadherin by TMEM16A, recombinant purified TGF-β was added at a concentration of 800 ng/ml to the culture medium of TMEM16A knockdown group. Supplement of TGF-β significantly reverted the upregulation of E-cadherin by knockdown TMEM16A (Fig. 8F, S3F). Furthermore, supplement of TGF-β significantly reverted the suppressive effects of TMEM16A knockdown on the migration and invasion of AGS and BGC-823 cells (Fig. 8G, S3G). The results revealed that knockdown of TMEM16A prevented AGS and BGC-823 cells migration and invasion by impairing TGF-β secretion to upregulate E-cadherin.

## DISCUSSION

Overexpression of TMEM16A is thought to be involved in carcinogenesis and tumor progression in multiple studies. In this study, the oncological significance of TMEM16A for GC was investigated and it was demonstrated that (1) TMEM16A was significantly upregulated and amplified in GC and lymph node metastases, (2) high level of TMEM16A in primary tumor associated with lymph node metastasis, higher disease grade and poor prognosis, (3) TMEM16A contributed to invasion rather than proliferation of GC cells, (4) TMEM16A expression was negatively correlated with E-cadherin expression in GC tissues, while positively correlated with serum levels of TGF-β1 from GC patients, (5) TMEM16A served as driving force to promote the secretion of TGF-βs, which suppressed E-cadherin expression to facilitate GC progression.

Before recognized as CaCC channel, TMEM16A had been found to be a tumor marker of GIST [26]. Recently, functional role of TMEM16A in other cancers was explored. In esophageal squamous cell carcinoma (ESCC) and prostate cancer, TMEM16A overexpression is positively correlated with lymph node metastasis and higher TNM stage [7, 8, 27]. In breast cancer, TMEM16A overexpression is negatively predictive for overall survival. Christian Ruiz et al. reported that high level of TMEM16A in primary tumor showed a significant trend towards presence of lymph node metastases by analysis 242 HNSCC specimens [7]. Consistent with our data, these findings regarding the oncogenic role of TMEM16A in different malignant tumors suggest that TMEM16A may ubiquitously promote carcinogenesis. However, it may seem controversial whether TMEM16A expression in the primary tumor differs from metastatic nodal tissue. In the present study, we found TMEM16A was similarly overexpressed in 30 GC primary tumor/metastatic lymph node pairs. In contrast, Daniel J et al. recently found that TMEM16A was overexpressed in primary HNSCC but downregulated in lymph node metastases, and TMEM16A expression in the primary HNSCC did not associated with the development of nodal metastases [28]. It should be noted that the number of HNSCC primary tumor/metastatic lymph node pairs in Daniel's study was relatively small, and whether clinical specimens collected before or after therapy were unknown since therapeutic stress such as radiotherapy and chemotherapy might affect TMEM16A expression. Also the possibility that TMEM16A is modulated by particular organ environments can not be ruled out. Further investigations are needed to determine if TMEM16A expression varies between primary and metastatic lesions of lymph node and distal organs.

TMEM16A is located on 11q13 which is frequently amplified in different cancers, and 11q13 amplification may be one reason for TMEM16A overexpression [7, 9]. In our study, TMEM16A overexpression was more pervasive than amplification in GC tissues. It is postulated that overexpression of TMEM16A might be driven by alternative regulatory mechanisms such as mTOR signalling, histone deacetylase [29, 30]. In fact, similar discrepancy is not uncommon for oncogenes amplification and overexpression. For instance, although CCND1 amplification contributes to CyclinD1 overexpression in breast cancer, high levels of CyclinD1 might result from estrogen receptor [31].

Different labs found that TMEM16A promoted proliferation in a variety of cancer cell lines, such as HNSCC(CAL33), breast cancer (MCF10A), pancreatic cancer (CFPAC-1), and prostate cancer (PC-3) [32]. However, a significant effect of TMEM16A knockdown on the proliferation of GC cells was not found in this study. Our findings were consistent with a previous report that downregulation of TMEM16A did not affect cancer cell growth [29], such as HNSCC cell line BHY [7]. Interestingly, a recent study in vascular smooth muscle cells suggested TMEM16A served as a negative regulator of proliferation as knockdown of TMEM16A facilitated and overexpression of TMEM16A inhibited angiotensin-II-induced cell cycle transition and cell growth [33]. In addition, TMEM16A may be inhibitory on proliferation of mouse intestinal epithelia cells [29]. Moreover, Chloride channel activity of TMEM16A was required for viability and proliferation [6, 7, 10]. Anke Bill et al. recently found influence of CaCCinh-A01 on cell viability depended on TMEM16A amplification and overexpression [34]. However, it should be noted that T16A-inh01, another chloride conductance inhibitor of TMEM16A, induced reduction in cancer cell viability regardless of TMEM16A amplification in Umamaheswar Duvvuri's study [6]. The discrepancy between the results regarding TMEM16A in cell proliferation appears to reflect the distinct functions of the same gene in various cell lines. Also it should be noted that TMEM16A could undergo alternative splicing, specific isoforms have been found in human diseased tissues. The expression and function of TMEM16A is tissue dependent and regulated by alternative splicing [24].

Molecular signalling regulating tumor metastasis is not clear. We found that TMEM16A promoted GC migration and invasion by modulating TGF-βs secretion. However, other molecular mechanisms might also be involved, which may explain that supplementation of TGF-βs did not completely revert the effects mediated by TMEM16A knockdown. Previous findings indicated that TMEM16A regulated cell shape and volume to facilitate diapedesis [29], and interacted with ezrin-radixin-moesin network [35], which contributed to cell movement and metastasis. In addition, a two hybrid split ubiquitin screening suggested that TMEM16A interacted with attachment proteins, such as catetnin [29], by which E-cadherin connects to actin as to maintain polarity and stability of epithelial cells and inhibit motility. Moreover, recent studies revealed TMEM16A served as important regulator of oncogenic signaling such as mitogen-activated protein kinase (MAPK) [6], sonic hedgehog [29], calmodulin-dependent protein kinase (CAMK) and epidermal growth factor receptor (EGFR) signaling pathway [9]. Interestingly, in cancer development TGF-βs signaling interacted with oncogenic signaling such as MAPK, PI3K-Akt [16]. Also it was found that membrane depolarization activated PI3K-Akt pathway [36], and changes in intracellular ion homeostasis regulated EGFR signaling [37]. Taken together, our study suggested that TGF-βs might serve as an intermediator between TMEM16A and oncogenic signaling such as MAPK. Activation of TMEM16A chloride channel depolarized membrane potential, promoted TGF-βs secretion, downregulated E-cadherin expression and facilitated GC migration and invasion. TGF-β is found to be a potent secreted cytokine that drives cancer progression, not only through its immunosuppressive and proangiogenic roles, but also perhaps more importantly as a potent inducer of epithelial-to-mesenchymal transition by regulating E-cadherin expression [16]. Consistent with the recent study [18], we found the TGF-β1 and TGF-β2 of serum from GC patients were significantly increased. The source of elevated TGF-β1 and TGF-β2 of serum is unclear. Our study suggested autocrine and paracrine TGF-βs from cancer cells might be an important source. Evidences implicate that high levels of TGF-β1 and TGF-β2 promote tumor cell local invasion and distal metastasis of tumor by the means of autocrine and paracrine mechanisms [21, 38]. Besides TGF-βs, TMEM16A is found to modulate the secretion of other factors such as mucin, interleukin-6/8, and CXCL1/2 [20, 22, 23]. Whether these factors involved in the regulation of tumor metastasis by TMEM16A needs further investigation.

## MATERIALS AND METHODS

### Patients

A total of 367 primary GC tissues, 30 corresponding lymph node metastatic lesions and 20 adjacent no-tumor tissues were recruited in the present study. All these patients underwent initial surgical resection and were pathologically confirmed as gastric adenocarcinoma in the First Affiliated Hospital of Sun Yat-sen University from January 2002 to October 2006. All follow-up information was completed (range from 1 to 62 months). The gender ratio was 2.3:1 (male: female). The median age was 57 years (range from 25–88 years). Furthermore, they had no chemotherapy, radiation therapy and surgery history. Routine chemotherapy was given after resection of primary gastric tumors and no radiation treatment was administered to any of the patients. Additionally, 5 mounted section tissues were recruited for this study. Moreover, 105 patients with GC had blood samples collected for the plasma measurement of TGF-βs. Prior patients' informed consent and the Institute Research Medical Ethics Committee of Sun Yat-sen University granted approval for this study.

### Tissue microarray construction

We firstly re-reviewed hematoxylin and eosin-stained slides, and selected the tumor zone in the paraffin-embedded specimens for tissue microarray (TMA) construction. Tissue microarrays were constructed in accordance with a previously described method [39]. For each case, two core taken from the selected tumor area and additional one core from adjacent non-tumor mucosa were used to construct the TMA. Briefly, a hollow needle was utilized to punch and remove bipartite cylinders tissue core (1.0 mm in diameter) from selected donor tissue regions. Further, the punched tissue cores were inserted into a recipient paraffin block with a precisely spaced array pattern, using an automatic tissue arraying instrument (Beecher Instruments, Silver Spring, Maryland, USA).

### Immunohistochemistry analysis

Immunohistochemical (IHC) analysis was done to study altered protein expression in 367 human GC tissues, 20 normal adjacent mucosal tissue controls, and 30 lymph node metastases. Besides, 5 mounted sections, which containing cancer tissue, tumor-adjacent tissues and normal mucosal tissues in one slide simultaneously, were also detected by IHC. TMA slides and mounted sections were incubated at 4°C in a moist chamber overnight with rabbit polyclonal antibody against human TMEM16A (1:500, Abcam, ab53212) and E-cadherin (1:500, Abcam, UK). A GC specimen was stained with PBS instead of primary antibody against TMEM16A or E-cadherin and this was used as negative control.

### Semi-quantitative assessment of immunohistochemical staining

Protein expression levels of TMEM16A or E-cadherin were evaluated by microscopic examination of stained tissue slides. Brown membranous immunoreactivity for the TMEM16A or E-cadherin protein were regarded as positive staining. TMEM16A or E-cadherin expression level was evaluated by integrating the percentage of positive tumor cells and the intensity of positive staining according to a previously described method [40]. The intensity of staining was scored as follows: negative (score 0), bordering (score 1), weak (score 2), moderate (score 3), and strong (score 4). We scored the staining extent according to the percentage of positive stained cells in the field: negative (score 0), 0–25% (score 1), 26–50% (score 2), 51–75% (score 3), and 76–100% (score 4). The sum of the intensity and extent score was considered as the overall IHC score (values from 0 to 16). Results were observed and assessed by two independent pathologists, without knowing the identity of the samples.

### Selection of cutoff score for each biomarker “positive” expression

The receiver operating characteristic (ROC) curve analysis was used to selection of cutoff score in the training set as previous study reported [41]. Briefly, the sensitivity and specificity for patient outcome at each score were plotted to generate a ROC curve. The score localized closest to the point at both maximum sensitivity and specificity, i.e. the point (0.0, 1.0) on the curve, was identified as the cutoff score that could be correctly classified patient outcome as death or alive.

### Fluorescence *in-situ* hybridization (FISH) studies

FISH studies were carried out on GC TMA and cells grown on coverslips. TMA was deparaffinized with xylene, dehydrated in 100% ethanol at room temperature, and incubated in 20 ug/ml protease K(Vysis) at 55°C for 10 min, followed by a wash in 2 × SCC for 5 min. Subsequently, slides were dehydrated in an ascending ethanol series (70, 80, 95%) and air-dried, and were heated to 78°C for 8 min. A premixed hybridization cocktail containing 5 μl CEP11 labeled with SpectrumGreen (Abbott Molecular), 5 μl BAC clone (RP11–203N8) labeled with SpectrumOrange, and 15 μl hybridization buffer (Vysis) was denatured at 78°C for 8 min. Hybridization was then performed by applying probe mixture to slides overnight at 37°C. The next day, slides were washed in 50% formamide/2 × SCC, 2 × SCC at 43°C for 5 min. Nuclei were counterstained with DAPI (Vector Laboratories, Burlingame, CA). The slide was viewed under a fluorescence microscope. TMEM16A was defined as amplified when signals were at least twice as common as centromere 11.

### Cell lines and cell culture

Human gastric adenocarcinoma AGS cells (ATCC, Manassas, VA, USA) were grown in F-12k (ATCC). Other GC cells MKN-45, BGC-823, SGC-7901, MKN-28 and normal human gastric epithelial cell line GES-1 (Shanghai Institute of Cell Biology, China) were grown in RPMI-1640 (Gibco). All of these cells were supplemented with 10% fetal bovine serum and 1% penicillin-streptomycin at 37°C with humidified 5% CO_2_.

### Stable short hairpin RNA transfection

Short hairpin RNA (shRNA) lentivirus for human TMEM16A(NM_018043) was from Sigma-Aldrich (MISSION shRNA Lentiviral Transduction Particles TRCN0000040263). GC cells were grown to 80% confluence and then infected with 1 multiplicity of infection of either non-targeting scrambled shRNA (SHC002V) control particles (SCR) or TMEM16A shRNA lentiviral particles (ShTM) in medium containing 8 ug/mL polybrene. Culture medium containing 4 μg/mL puromycin was added after 48 hours to select for puromycin-resistant cells.

### Quantitative real-time polymerase chain reaction (qRT-PCR)

Total RNA was isolated using TRIzol reagent (Invitrogen, USA). Complementary DNA was prepared using oligo^dT^ primers according to the protocol supplied with the Primer Script TM RT Reagent (TaKaRa, Japan). Expression of TGF-β1 and TGF-β2 was determined by quantitative real-time PCR using Power SYBR green PCR master mix (Applied Biosystems) and primers were shown in Table S3.

### Western blot

Cells and Liquid nitrogen-conserved gastric cancer tissues (4 pairs of gastric cancer and matched adjacent non-tumor specimens from 4 patients randomly selected) were ground and lysed with the RIPA buffer on ice. Protein concentration was determined by the Bradford method with bovine serum albumin as the control. Equal amounts of tissue lysates (50 μg) were run by SDS-PAGE, and electro-transferred on a polyvinylidene difluoride membrane. The membrane was then blocked and incubated with primary antibodies against TMEM16A (1:500, Abcam, UK), β-actin antibody (1:1000, Santa Cruz Biotechnology, CA) and E-cadherin (1:500, Abcam, UK), TGF-β1 and TGF-β2 (1:400; Santa Cruz Biotechnology, CA), respectively, for 2 hour at room temperature, and then incubated with appropriate horseradish peroxidase-conjugated secondary antidodies (1:1000, Cell Signaling Technology) for 1 hour at room temperature. Final detection was carried out with LumiGLO chemiluminescent reagent (New England Biolabs) as described by the manufacturer. The densities of target bands was accurately determined by the computer-aided 1-D gel analysis system.

### Cell proliferation assay

Cell proliferation was assessed by cell counts and 5-bromo-2′-deoxyuridine (BrdU) incorporation as described previously [42]. For the cell counts, GC cells (CTR), GC cells stably expressing TMEM16A shRNA (ShTM) or scrambled shRNA (SCR) were serum free for 24 h. Then cells were trypsinized and equal number (2 × 10^5^) of cells from each group was plated into 6-well culture plates in complete culture medium for 24, 48, 72 h. Then the cell number was determined in triplicate using a hemocytometer. BrdU incorporation was examined using 5-Bromo-2′-deoxy-uridine Labeling and Dectection kit III (Roche Applied Science, Mannheim, Germany) according to the manufacturer's instructions. Briefly, GC cells (CTR), stable expressing TMEM16A shRNA GC cells (ShTM), stable expressing scrambled shRNA GC cells (SCR) were serum free for 24 h. Then cells were trypsinized and equal number (2 × 10^4^) of cells from each group was plated into a 96-well plate and grown in complete culture medium with 10 μM BrdU for 2, 4 or 8 h. BrdU incorporation into cellular DNA was measured using a microplate reader (Safire II; Tecan, Mannedorf, Switzerland). Each assay was done in triplicate. The experiments were performed six times independently.

### Cell migration and invasion assay

Cell migration was assessed by wound healing assays and transwell assays. For the wound healing assay, AGS cells (CTR), AGS cells stably expressing TMEM16A shRNA (ShTM) or scrambled shRNA (SCR) were serum free for 24 h. Then cells were trypsinized and equal number (3.5 × 10^5^) of cells from each group was plated into 6-well culture plates in complete culture medium for 4 h, then a scratch lesion was created using a 200 μl pipette tip. To eliminate dislodged cells, culture medium was removed and wells were washed mildly with PBS. Then cells were grown in complete culture medium for 48 h until the digital images of cells migrated into the scratch were taken on an inverted microscope. Measurement of wound area was done using the Adobe Photoshop software. Wound closure was quantified as the mean ± standard deviation of three independent experiments. The control wound closure was set as 100%, and the wound closure of ShTM or SCR group was represented as the percent of the control. Transwell inserts with 8 μm pores (BD Biosciences, San Jose, CA, USA) for transwell migration assays, 2 × 10^5^ cells in serum free medium were added to each upper compartment of the chamber. After 48 h incubation, noninvasive cells were removed from the upper surface of the transwell membrane, and migrated cells were fixed with methanol, stained with 1% crystal violet and counted using a light microscope in 5 random visual fields at the magnification of 100X.

### Current recording

TMEM16A Ca^2+^-activated Cl^−^ current was recorded with an Axopatch 200B Amplifier (Axon Instrument, Foster City, CA) with a whole-cell patch clamp technique as previously described [42]. The currents were elicited with voltage steps from −100mV to +120 mV in + 20mV increment for 250 ms with an interval of 5s from a holding potential of −50mV. Currents were sampled at 5 kHz using pCLAMP8.0 software (Axon Instruments) and filtered at 2 kHz. Extracellular solution contained (mM): NMDG Cl 125, KCl 5, CaCl2 1.5, MgSO4 1, HEPES 10, Glucose 10, pH was adjusted to 7.4 with NMDG. Pipette solution contained (mM): CsCl 130, Mg ATP 1, MgCl2 1.2, HEPES 10, EGTA 2, and CaCl2 1.639, pH was adjusted to 7.3 with CsOH. The intracellular Ca^2+^ concentration ([Ca^2+^]i) was 500 nM.

### Enzyme-Linked Immunosorbent Assay (ELISA)

TGF-β1 and TGF-β2 levels were determined by sandwich ELISA using Quantikine human TGF-β1 immunoassay and TGF-β2 immunoassays (R&D Systems, USA) according to the manufacturer's instructions. Briefly, 100 μl of cell supernatants or 40 μl of serum, were treated with 20 μl of 1M HCl for 10 min, then neutralized by 20 μl of NaOH/0.5MHEPEs. The samples were pipetted into microplate wells precoated with a monoclonal antibody specific for TGF-β1 or β2, and incubated for 2 h at room temperature. An enzyme-linked polyclonal antibody specific for TGF-β1 or β2 was then added to the wells and incubated for a further 2 h to sandwich the TGF-β1 or β2 ligand. A substrate solution was added and the intensity of the color was determined using an automicroplate reader (Flexstation 3, Molecular Devices). Each experiment was conducted twice and each sample point was assessed in triplicate.

### Statistical analyses

All statistical analyses were performed using SPSS 19.0 statistics software. For survival analysis, 113 cases were randomly assigned to the training set to generate the immunohistochemical optimal cutoff point of TMEM16A proteins by receiver operating characteristic (ROC) analysis. For validation, the relationship between TMEM16A expression, which was classified by ROC analysis-generated cutoff point, and overall survival(OS) were evaluated in the testing set (*n* = 254) and overall patients (*n* = 367). The chi-square test or Fisher's exact test was carried out to evaluate the relationship between TMEM16A and clinicopathological variables. The relationship between TMEM16A expression and OS was determined by Kaplan-Meier analysis, and differences in survival probabilities between patient subsets were assessed by the log-rank test. The multivariate Cox proportional hazards model was utilized to determine independent prognostic factors. The correlations between TMEM16A expression and amplification, expression of TMEM16A and E-cadherin were assessed by Phiand Cramers V correlation analysis. Statistical analysis of cellular proliferation, migration and invasion was performed using ANOVA or Student's unpaired *t*-test. Data from all quantitative assays were expressed as the mean ± standard and values of *P* < 0.05 were considered statistically significant.

## CONCLUSIONS

In conclusion, our study provided both clinical and experimental evidence that TMEM16A acts as an oncogene in GC. TMEM16A modulates cellular invasiveness at least in part via modulating TGF-βs secretion.

## SUPPLEMENTARY FIGURES AND TABLES


