# Supplementary material for: TMEM16A overexpression contributes to tumor invasion and poor prognosis of human gastric cancer through TGF-β signaling
Source: Oncotarget. 2015 Mar 23;6(13):11585–99. doi: 10.18632/oncotarget.3412 (PMC4484478; doi:10.18632/oncotarget.3412)
Supplement: Supplementary file 1 [file oncotarget-06-11585-s001.pdf]

SUPPLEMENTARY FIGURES AND TABLES

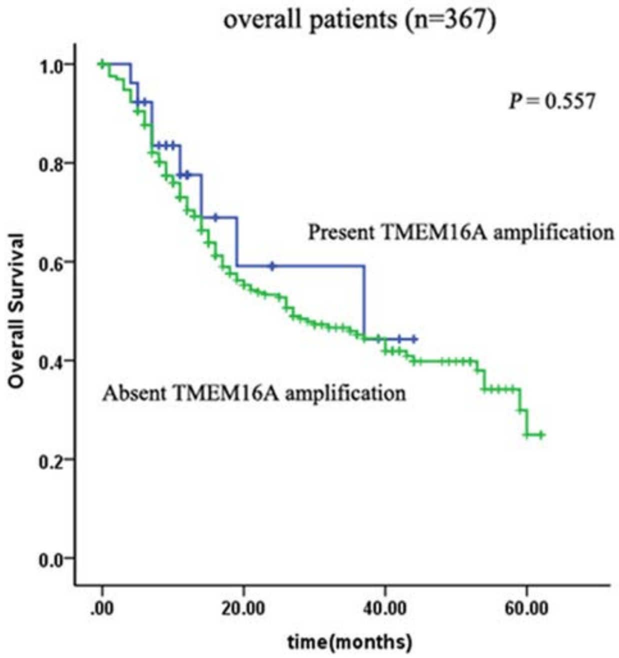

Supplementary Figure S1: Kaplan–Meier estimated of overall survival according to TMEM16A amplification in patients with gastric cancer (log-rank test). Amplification of TMEM16A was not significantly correlated with overall survival ($p = 0.557$).

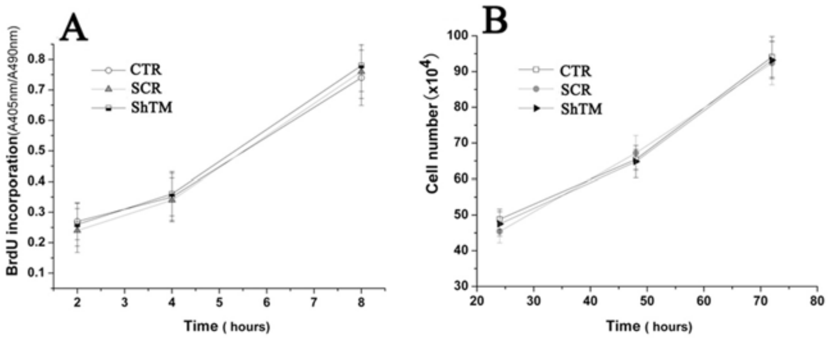

Supplementary Figure S2: Knockdown of TMEM16A does not affect BGC-823 cell proliferation. A. BrdU incorporation assay at 2 hours, 4 hours and 8 hours and **B.** cell count experiments at 24 hours, 48 hours and 72 hours from CTR group, SCR group and ShTM group ($n = 6$, $p > 0.05$).

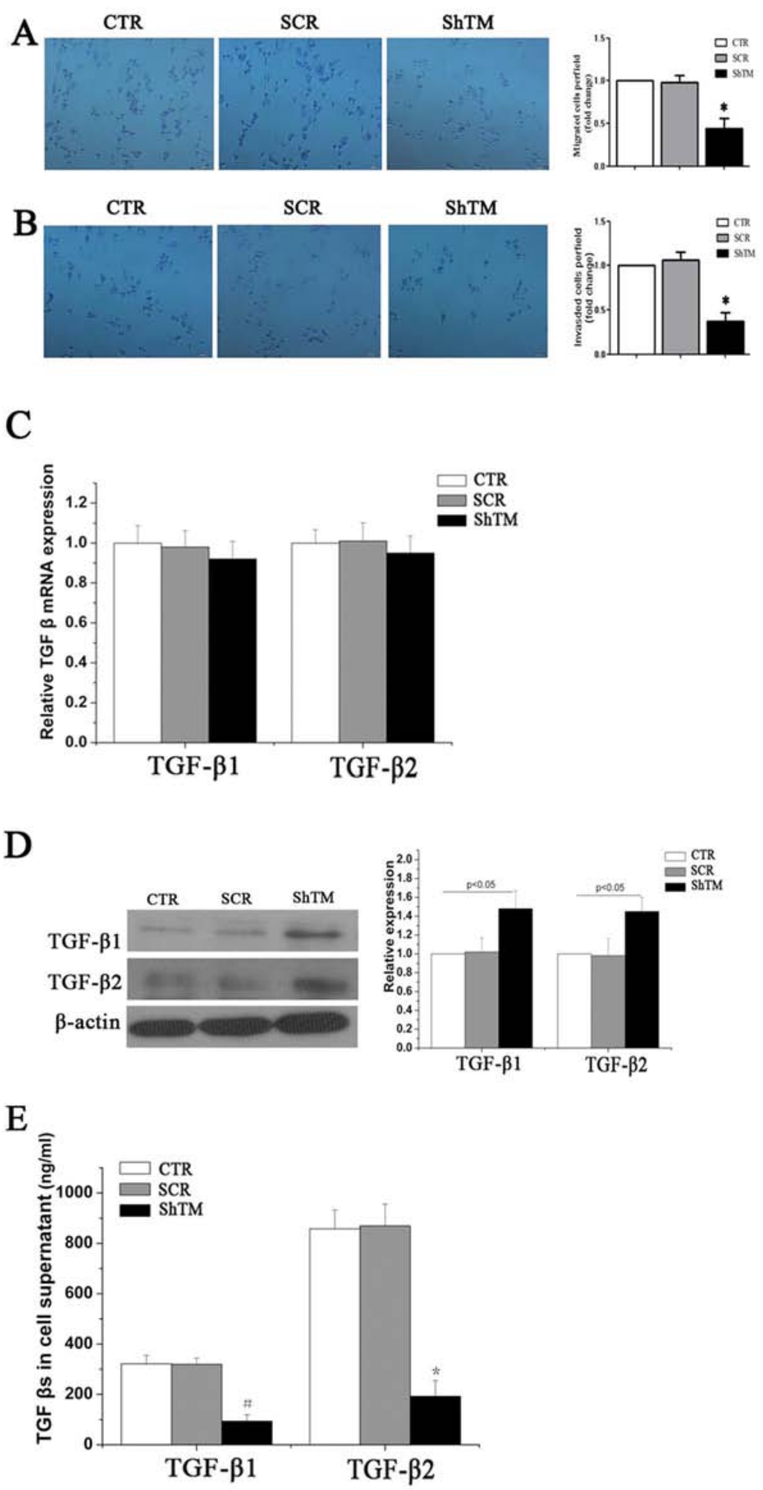

Supplementary Figure S3: Knockdown of TMEM16A prevents the invasion and migration of BGC-823 cells by TGF-βs signalling. **A.** The transwell migration assay and **B.** transwell invasion assay revealed TMEM16A knockdown reduced migration and invasion ($n = 6$, $*p < 0.05$ vs CTR group). **C.** Knockdown of TMEM16A did not affect mRNA expression of TGF-β1 and TGF-β2 in BGC-823 cells ($n = 6$, $p > 0.05$). **D.** Protein expression of TGF-β1 and TGF-β2 in BGC-823 cells increased when knockdown of TMEM16A as shown by western blot analysis ($n = 6$, $p < 0.05$ vs CTR group). **E.** Supernatant concentrations of TGF-β1 and TGF-β2 dramatically reduced when knockdown of TMEM16A as shown by ELISA ($n = 6$, $^{\#}p < 0.01$ vs CTR group, $*p < 0.05$ vs CTR group).

(*Continued*)

F

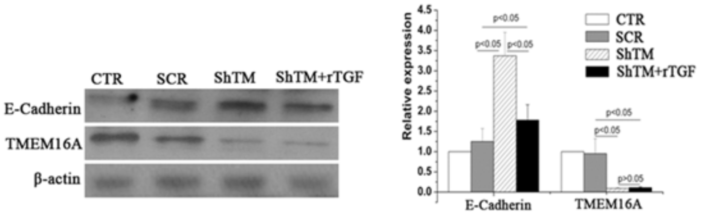

G

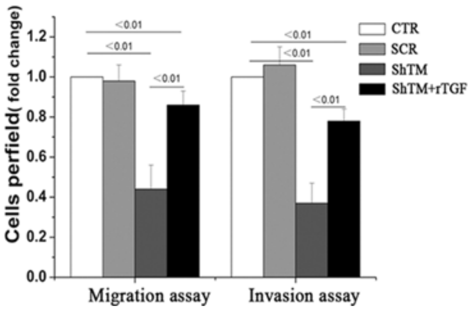

Supplementary Figure S3 (*Continued*): E. Supernatant concentrations of TGF-β1 and TGF-β2 dramatically reduced when knockdown of TMEM16A as shown by ELISA (n = 6, $^{\#}p < 0.01$ vs CTR group, $*p < 0.05$ vs CTR group). **F.** Adding recombinant purified TGF-β increased E-cadherin expression in BGC-823 cells with TMEM16A knockdown(n = 6). **G.** Supplement of recombinant purified TGF-β increased the migration and invasion of cells with TMEM16A knockdown (n = 6). CTR: control BGC-823, SCR: scrambled BGC-823, ShTM: shTMEM16A BGC-823.

Supplementary Table S1. The correlation between TMEM16A expression and amplification in gastric cancer tissues

Variables		All cases	TMEM16A expression		*P* value	Phi
			High(%)	Low(%)		
**TMEM16A amplification**	Absent(%)	340	230(67.6%)	110(32.4%)	0.021	0.120
	Present(%)	27	24(88.9%)	3(11.1%)		

Supplementary Table S2. Correlation of TMEM16A amplification and TMEM16A expression with patients' features in gastric cancer

Variables	All cases	TMEM16A amplification (overall patients n = 367)			TMEM16A expression (overall patients n = 367)		
		Absent	Present	*P* value[a]	Low	High	*P* value[a]
**Gender**							
Male	256	235	21	0.346	74	182	0.235
Female	111	105	6		39	72	
**Age at surgery**							
≥57[b]	191	174	17	0.238	51	140	0.077
<57	176	166	10		62	114	
**Tumor location**							
Upper half	193	179	14	0.484	57	136	0.695
Lower half	158	145	13		52	106	
Whole	16	16	0		4	12	
**Tumor size**							
≥5cm[c]	221	205	16	0.916	66	155	0.636
<5cm	146	135	11		47	99	
**Histological type**							
Intestinal	288	267	21	0.927	84	204	0.198
Diffuse	79	73	6		29	50	
**TNM**							
I + II	139	121	18	**0.001**	61	78	**0.000**
III + IV	228	219	9		52	176	
**Lymphnode metastasis**							
Present	249	236	13	**0.023**	57	192	**0.000**
Absent	118	104	14		56	62	

[a]chi-square test
[b]median age
[c]median tumor size

Supplementary Table S3. Primers used for real-time PCR

Gene and primer		Sequences(5′ to 3′)
NM_000660.4	TGF-β1_F	CCCACAACGAAATCTATGACAA
	TGF-β1_R	AAGATAACCACTCTGGCGAGTC
NM_001135599.2	TGF-β2_F	ATTGCCCTCCTACAGACTTGAG
	TGF-β2_R	CAGCACAGAAGTTGGCATTGTA
NM_002046.3	GAPDH_F	GCACCGTCAAGGCTGAGAAC
	GAPDH_R	TGGTGAAGACGCCAGTGGA
